# The Modification and Self-Lubricating Properties of CNTs-Enhanced PEEK Porous Composites Based on FDM

**DOI:** 10.3390/nano15181411

**Published:** 2025-09-13

**Authors:** Zhuangya Zhang, Baorun Yang, Xiaoqiang Wang, Ruijie Gu, Mingde Duan

**Affiliations:** School of Mechanical and Electrical Engineering, Henan University of Science and Technology, Luoyang 471000, China

**Keywords:** carbon nanotubes (CNTs), PEEK, porous, FDM, tribological properties

## Abstract

Porous composites utilize their unique pore structures to effectively store and release lubricants, providing a fundamental mechanism for continuous lubrication in self-lubricating bearing cages. This study investigates carbon nanotube (CNT)-reinforced PEEK porous composites fabricated by fused deposition modeling (FDM) and subsequently subjected to heat treatment to improve tribological properties. Results show that incorporating 3 wt% CNTs significantly enlarges average pore size from 0.08 μm to 11.62 μm and increases porosity, resulting in an oil retention rate exceeding 80%. The composites achieve a 26.4–63.4% reduction in friction coefficient under dry sliding conditions at room temperature. After heat treatment, the material maintains a stable friction coefficient below 0.30 during high-temperature dry friction, demonstrating excellent lubricant slow-release capability and thermal stability.

## 1. Introduction

As critical supporting components, bearings and bearing units directly determine the operational lifespan of equipment. The rapid development of fields such as high-speed rail, aviation, and aerospace has led to the performance of high-end motion evolving towards more severe and extreme conditions [1]. Studies indicate that lubrication failure is a major cause of bearing malfunctions. For instance, dozens of satellite failures have been attributed to bearing lubrication failures [2]. In 2005, an aircraft accident was traced to spindle bearing raceway wear and cage fracture, ultimately leading to seizure failure [3]. The lubrication of bearings in high-end equipment presents unique challenges. it cannot be supplemented with lubricating oil. Therefore, the special working conditions of the aerospace bearing determine that it cannot adopt conventional lubrication technology, and the lubrication problem limits the service performance in extreme environments.

Porous self-lubricating bearings offer a promising solution to the aforementioned challenges. When lubricating oil or grease is absorbed into the pores of a porous cage, a self-lubricating effect can be achieved during operation under the influence of centrifugal force, temperature rise, and other factors, thereby reducing friction and wear [4,5,6]. Common materials for porous self-lubricating bearings in space applications include cloth-phenolic materials (nylon or PA), polyimide (PI), and polyetheretherketone (PEEK) [7]. although phenolic materials have been widely used historically, their strong hygroscopic nature significantly compromises oil retention capacity. In contrast, PI suffers from poor melt-processability, making it difficult to fabricate. PEEK, a semi-crystalline high-performance thermoplastic polymer, has been extensively employed in aerospace, medical devices, and the energy sector due to its excellent mechanical properties, high-temperature resistance, and chemical stability. Moreover, its outstanding processability and tribological characteristics make PEEK an ideal material for porous bearing [8,9,10,11]. The research on PEEK tribological modification of polymers and composite materials shows the following findings: Rodriguez et al. [12] found that pure PEEK matrix material exhibits a low wear rate under dry friction conditions but has a higher friction coefficient. Adding fiber-reinforced materials and lubricants can significantly reduce the friction coefficient, but its wear behavior shows some uncertainty. Samyn et al. [13,14] discovered that filling with polytetrafluoroethylene (PTFE) effectively reduces the friction coefficient of carbon fiber-reinforced thermoplastic polyimide (CF-TP), while CF-TP composites filled with silicone oil promote the formation of a uniform lubricating film under high loads, improving their wear resistance. Pozdnyakov et al. [15] investigated the structural and compositional evolution of PMMA–fullerene C_60_ and PMMA–multiwalled carbon nanotube composites under different heat treatment conditions by comparing their NEXAFS and TD-MS spectra. Koberni et al. [16] proposed a plasma surface cladding process modified with multi-walled carbon nanotubes (MWNTs). Their experimental results demonstrated that adding 0.25 wt% MWNTs significantly enhanced friction stability, reduced the coefficient of friction, and improved wear resistance by approximately 5%. Microstructural analysis confirmed that the MWNTs remained stable during cladding and were effectively incorporated into the coating, providing a mechanistic explanation for the enhanced tribological performance. Liu et al. [17] investigated the effect of microsphere fillers on the tribological properties of short carbon fiber (SCF)-reinforced PEEK composites. The results show that under different load conditions, SCF-reinforced PEEK composites have both lower friction coefficients and better wear resistance compared to pure PEEK material, with the SCF-reinforced PEEK composites filled with 10 wt% microsphere fillers exhibiting the best tribological performance. Shang [18] studied the effect of multi-walled carbon nanotubes (MWCNTs) of varying lengths on the thermal, mechanical, and tribological properties of PEEK-based composites. The results indicated that MWCNTs in the length range of 10–30 μm help improve the crystallization temperature and mechanical properties of PEEK composites, while MWCNTs around 50 μm effectively reduce adhesive wear and friction coefficients, enhancing wear resistance. Yang YongXi [19] employed cold-press sintering to prepare porous PI materials and introduced carbon nanotubes (CNTs) for modification. The research found that CNTs have limited effects on the mechanical properties and friction coefficient of porous materials, but they can effectively enhance the oil content and oil retention. The friction coefficient showed a trend of initially decreasing, then increasing, and finally decreasing again with the increase in CNT content. At 0.2% CNT content, the friction coefficient was less than 0.1. Pang Xianjuan et al. [20] studied the effect of carbon fiber (CF) content on the properties of CF/PEEK composites prepared by vacuum hot pressing sintering. The results indicated that with the increase in CF content, the friction coefficient, wear rate, and friction electrostatic voltage of the composites initially decreased, then increased. When the CF content reached 20%, the friction coefficient, wear rate, and friction electrostatic voltage were at their minimum.

Currently, the mainstream preparation processes for porous self-lubricating bearing materials are cold pressing sintering and template-filtration methods. Fused Deposition Modeling (FDM) is one of the most commonly used printing technologies in additive manufacturing (also known as 3D printing). FDM technology constructs three-dimensional objects by depositing molten material layer by layer. This layer-by-layer construction approach allows for flexible adjustment of material composition and printing parameters during the manufacturing process, offering the possibility of customizing and optimizing high-performance composite materials [21,22,23]. Ata et al. [24] found that thermal treatment can significantly increase the crystallinity of composites, thereby enhancing their mechanical properties. They pointed out that thermal treatment promotes the reorganization of the molecular chains of polyetheretherketone (PEEK), forming a more stable crystalline structure, which in turn improves the tensile strength and elastic modulus of the composite materials. Hou Yuejiao et al. [25] conducted thermal treatment research on carbon nanotube (CNTs)-reinforced PEEK composites, and the results showed that the optimal thermal treatment time was 2 h. Thermal treatment significantly improved the tensile and flexural strengths of the composite materials by 9.2% and 18.6%, respectively, while the crystallinity increased by 4.88%. Ye Xin, in his thesis [26], the study found that after annealing treatment, the crystallinity of the composite material was significantly enhanced, with its tensile strength and elastic modulus increasing by 19.9% and 19.5%, respectively.

Although previous studies have separately investigated the reinforcing effect of CNTs on PEEK, the enhancement of mechanical properties through heat treatment, and the self-lubricating behavior of porous materials, a systematic examination of the structure–property relationship between microstructural evolution and tribological performance in additively manufactured CNT-reinforced porous PEEK composites after optimized heat treatment remains unexplored. The present study aims to address this research gap. Building upon the group’s earlier work [27,28,29], we synergistically integrate material design (CNT content), an innovative fabrication process (fused deposition-washing), and post-processing optimization (heat treatment) to establish a theoretical foundation for designing and manufacturing high-performance self-lubricating bearing cages with tailored structure–property integration.

## 2. Preparation of Composite Filaments

### 2.1. Preparation of Composite Filaments

To enhance the interfacial bonding between the CNTs and the matrix material and to prevent the agglomeration of CNTs within the porous structure (which could affect pore formation), the raw powders underwent a pretreatment process. This process included surface modification, particle size adjustment, and dispersion optimization.

PEEK powder (200 mesh, 1.3 g/cm^3^, melting point 334 °C) was supplied by Jilin Huana Special Polymer Co., Ltd. (Changchun, China) CNTs (TNMC7 type) were provided by Chengdu Zhongke Shidai Nanneng Technology Co., Ltd. (Chengdu, China), with key properties detailed in Table 1. NaCl powder (density 2.1 g/cm^3^, melting point 804 °C) was obtained from Shanghai Maclin Biochemical Co., Ltd. (Shanghai, China), and sieved through a 325 mesh sieve before use.

The pretreatment of the CNTs was conducted as follows: First, the CNTs were reflux-washed with acetone, rinsed with ethanol, and then dried at 120 °C to remove surface sizing agents and dust. Subsequently, the CNTs were subjected to ultrasonic dispersion for 5 h to improve their dispersion uniformity, followed by oven-drying for later use.

For the modification of nano-calcium carbonate (nano-CaCO_3_), the following procedure was employed: The nano-CaCO_3_ was first ultrasonically dispersed in deionized water for 30 min. The slurry was heated to 90 °C in a water bath, followed by the addition of molten sodium stearate and a 2 h reaction at the same temperature Afterwards, the mixture was filtered, and the resulting filter cake was washed with hot anhydrous ethanol, dried, ground, and sieved for subsequent use.

The specific mass ratios of PEEK/NaCl and the CNTs addition amounts are listed in Table 2. According to the formulations in Table 2, the required amounts of NaCl and PEEK powder (both pre-dried at 120 °C for 12 h) were accurately weighed. The pretreated CNTs were also weighed and further dried in an oven at 80 °C for 6 h. Based on the total mass of the PEEK/NaCl composite powder, 0.5 wt% and 1 wt% of cyclohexane, 1,2-dimethyl-2-isononyl ester, and the modified nano-calcium carbonate were weighed. The PEEK, CNTs, NaCl, and additive powders (cyclohexane, 1,2-dimethyl-2-isononyl ester, modified nano calcium carbonate) were mixed in a ball mill for 8 h. The well-mixed composite powder was then placed in an oven and dried at 120 °C for 10 h.

Filaments were prepared using a melt blending extrusion process. The temperatures of each zone of the twin-screw extruder were adjusted accordingly. Taking the preparation of the composite filament for sample CN3#-1 as an example, the temperature settings are provided in Table 3. The prepared filaments were stored in a 60 °C environment.

**Figure 1:**
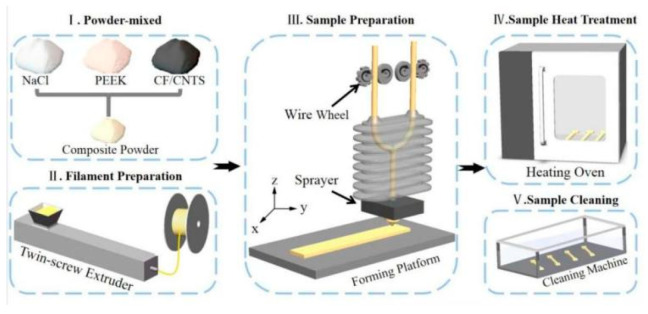
Preparation process of CNTS reinforced PEEK-based materials.

An FDM rapid prototyping machine (model: ENGINEER Q300, Shaanxi Jugaowang Additive Manufacturing Technology Development Co., Ltd., Weinan, China) was used for the additive manufacturing process. The key process parameters were set as follows: a nozzle diameter of 1.0 mm, a nozzle temperature of 420 °C, a printing speed of 30 mm/s, and a layer thickness of 0.2 mm (Figure 1).

The printed samples were placed in an ultrasonic cleaner for 48 h with electric stirring to leach out the internal porogen (NaCl). Subsequently, the samples were removed and dried at a low temperature in an oven for 5 h.

For the samples designated for heat treatment, they were placed in an oven with a controlled heating rate of 8 °C/min. The temperature was first raised to 260 °C and held for 90 min. It was then further increased to 300 °C at the same rate (8 °C/min) and maintained for 2 h. Finally, the samples were removed from the oven and allowed to cool naturally to room temperature.

### 2.2. Structural Characterization and Performance Testing

To explore the internal microstructure of CNTs-enhanced PEEK porous samples, a Laser Scanning Confocal Microscope (LSCM, Zeiss-LSM800, Oberkochen, Germany) was used to observe the microstructure of CNTs-enhanced PEEK-based composite materials; a Field Emission Scanning Electron Microscope (FESEM, EDS, JSM-IT800, Tokyo Electron, Tokyo, Japan) was used to test the NaCl removal effect inside the samples; The pore size distribution and porosity of the CNT-reinforced PEEK porous samples were characterized using a mercury intrusion porosimeter (Autopore IV 950, Micromeritics, Norcross, GA, USA) (Table 4).

Performance Testing: The macro-performance of the CNT-enhanced PEEK porous samples was evaluated using a high-temperature friction and wear tester (HT-1000, Lanzhou Zhongke Kaihua Technology Development Co., Ltd., Lanzhou, China) under dry sliding conditions. The tests were conducted under the following parameters: a 5 mm diameter 9Cr18 steel ball, 5 N normal load, 5 mm wear track radius, 392 rpm rotational speed, and 30 min test duration. The reported values represent the average of five independent measurements. A bearing cage vacuum oil impregnation machine (ZKXY-400, Luoyang Bearing Research Institute Co., Ltd., Luoyang, China) was used to press the lubricant into the sample, and the oil retention ability was measured by a centrifuge (TG16-WS, Hunan Xiangyi Laboratory Instrument Development Co., Ltd., Changsha, China). The oil content and oil retention ability were calculated using the specified methods.

(1) $$\mathrm{oil}\;\mathrm{content}=\frac{m_{0}-m_{0}^{\prime }}{m_{0}^{\prime }}\times 100\%$$

(2) $$\mathrm{oil}\;\mathrm{retension}=\frac{\mathrm{oil}\;\mathrm{content}\;\mathrm{of}\;\mathrm{the}\;\mathrm{sample}\;\mathrm{after}\;T\;\mathrm{minutes}\;\mathrm{of}\;\mathrm{oil}\;\mathrm{throwing}}{\mathrm{Original}\;\mathrm{oil}\;\mathrm{content}\;\mathrm{of}\;\mathrm{the}\;\mathrm{sample}}\times 100\%$$

*m*_0_′: weight of the CNTs-enhanced PEEK porous sample;

*m*_0_: weight of the oil-impregnated CNT/PEEK porous sample.

The sample is cleaned, weighed, and then immersed in lubricating oil. It is soaked under vacuum conditions (1.0 × 10^−3^ Pa) at a temperature of 70 °C for 48 h to allow the lubricating oil to fill the internal gaps. Subsequently, the sample was removed and drained at 25 °C for 48 h, then reweighed to determine its post-immersion mass. The oil-containing sample was then subjected to centrifugal treatment at room temperature (25 °C) for 2 h using a coaxial centrifuge operating at 8000 rpm. The mass of the sample before and after oil immersion under static conditions was measured, and the oil retention rate of the sample was calculated by Formula (2).

## 3. Results

### 3.1. Macroscopic Morphology of the Composite Filament

**Figure 2:**
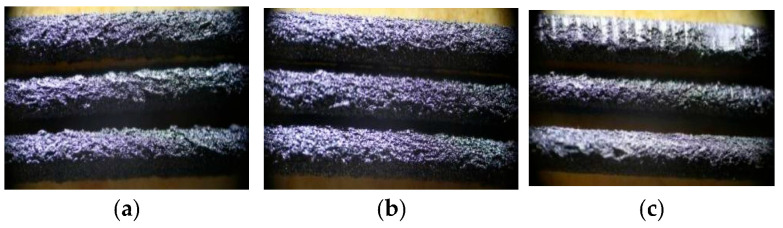
Surface morphology of composite filaments at different CNTs mass fractions (**a**) Sample CN3#-1; (**b**) Sample CN3#-2; (**c**) Sample CN3#-3.

**Figure 3:**
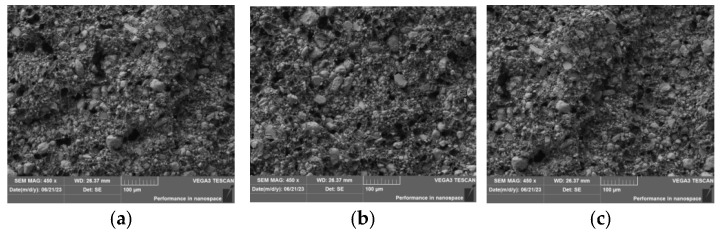
Fracture morphology of composite filaments at different CNTs mass fractions (**a**) Sample CN3#-1; (**b**) Sample CN3#-2; (**c**) Sample CN3#-3.

The composite filament’s surface morphology and fracture morphology were observed using an optical microscope (Figure 2 and Figure 3).

As illustrated in Figure 2, the surface roughness of the composite filaments progressively increases with the rising carbon nanotube (CNT) content. The incorporation of CNTs leads to noticeable deterioration in diameter control and winding performance of the filaments. This is attributed to the reduced flowability of the composite material and the agglomeration of CNTs at higher loadings, which collectively contribute to increased surface roughness and consequently impaired flexibility. As observed in Figure 3, the relatively uniform distribution of protrusions and pores on the fracture surfaces across various CNT mass fractions indicates effective interfacial adhesion between the CNTs and the PEEK matrix. This morphological homogeneity further suggests that the employed processing technique was successful in achieving a largely uniform dispersion of CNTs, thereby minimizing agglomeration-induced stress concentration.

### 3.2. Microscopic Morphology of Composite Filaments

**Figure 4:**
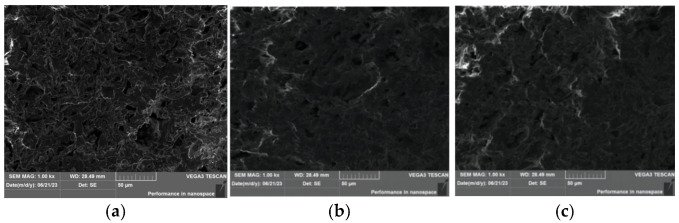
Microscopic morphology of porous materials (**a**) Sample CN3#-1; (**b**) Sample CN3#-2; (**c**) Sample CN3#-3.

**Figure 5:**
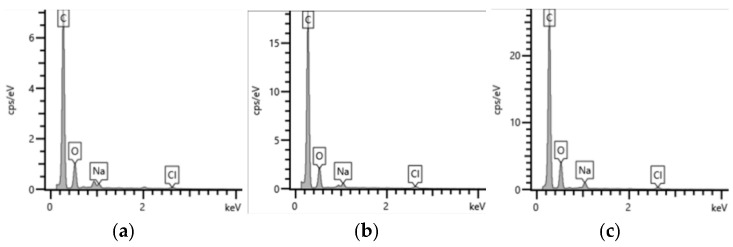
EDS analysis results of samples at different CNTs mass fractions (**a**) Sample CN3#-1; (**b**) Sample CN3#-2; (**c**) Sample CN3#-3.

The microstructure of carbon nanotube (CNT)-reinforced PEEK porous samples was characterized using laser confocal scanning microscopy and scanning electron microscopy. As revealed in Figure 4, the porous architecture exhibits a uniform distribution of pores with high inter-pore connectivity. This observation indicates that the introduction of CNTs did not adversely affect the pore-forming function of the NaCl porogen, suggesting good compatibility between the CNTs and the composite processing protocol. The EDS results presented in Figure 5 confirm the near-complete elimination of NaCl, as evidenced by the negligible residual chlorine and sodium signals. This efficient removal, achieved through aqueous leaching, is critical as it ensures that the tribological properties of the resulting CNT/PEEK porous composite are intrinsically attributable to its designed material composition and microstructure, free from the potential confounding effects of salt residues.

The SEM and EDS results indirectly indicate that CNTs achieved relatively uniform dispersion within the composite powder. Should severe agglomeration of CNTs occur, it would impede the homogeneous mixing of PEEK particles with NaCl particles, thereby leading to defects such as localized non-uniformity in the pore structure and reduced connectivity. No such defects were observed in this experiment, providing preliminary morphological evidence for the satisfactory dispersion of CNTs.

**Figure 6:**
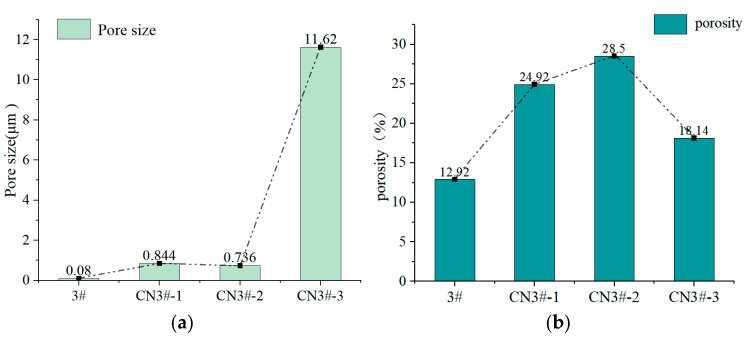
Characterization of microscopic pore parameters of porous samples (**a**) Pore size distribution of sample; (**b**) Porosity distribution of sample.

Figure 6 presents the mercury porosimetry results detailing the microstructural characteristics—specifically pore size and porosity—of the porous PEEK materials with and without CNT incorporation. The addition of CNTs markedly increased the average pore size. For instance, at a CNT loading of 3 wt%, the mean pore size increased dramatically from 0.08 μm (pristine PEEK) to 11.62 μm. The incorporation of CNTs impaired the flow properties of the composite melt, resulting in compromised fluidity that was insufficient to effectively separate NaCl particles. During mixing and processing, the NaCl particles exhibited a tendency to agglomerate, leading to the formation of larger aggregates. In the subsequent water-washing step, these NaCl agglomerates were completely leached out, leaving behind oversized pores corresponding to their dimensions. As a result, the average pore size increased markedly from 0.08 µm in pure PEEK to 11.62 µm.

As shown in Figure 6b, the porosity of the CNT-reinforced PEEK samples also rose significantly with the incorporation of CNTs. This increase is primarily due to two contributing factors: the creation of micron-sized pores by the NaCl porogen, and the additional pore interconnectivity facilitated by the hollow tubular structure of CNTs, which helps establish a continuous three-dimensional porous network. However, when the CNT content reached 3 wt%, the porosity decreased to 18.14%. This reduction suggests a threshold beyond which excessive CNT addition adversely affects material microstructure. The decline is likely caused by severely impaired melt flowability, which compromises the dispersion of both NaCl and CNTs, promotes agglomeration, and reduces pore connectivity. These changes manifest as a drop in measurable porosity, underscoring the dual role of CNTs in both enhancing and—at higher concentrations—diminishing structural porosity. This demonstrates that there exists an optimum addition point for CNTS content.

Although this phenomenon is detrimental to performance, it conversely demonstrates the sensitivity of dispersion state to CNT content, further confirming that CNTs indeed achieve relatively uniform dispersion at the aforementioned lower concentrations.

### 3.3. Oil Content and Oil Retention of the Material

**Figure 7:**
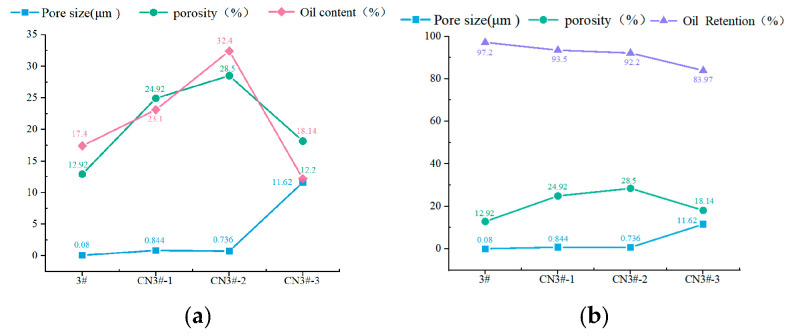
Oil content and oil retention rate test results of porous PEEK samples (**a**) Oil content; (**b**) Oil Retention.

Oil content and oil retention rate are important technical indicators for evaluating oil-containing materials. Five samples of each material type were tested; the oil content and oil retention rate test results of each porous PEEK sample are shown in Figure 7.

As seen in Figure 7, compared to pure PEEK porous samples, the oil content of the 1 wt% and 0.5 wt% samples is higher. The oil content of porous samples exhibits a highly consistent trend with changes in their porosity. Continuing to increase the CNTs content may cause CNTs to agglomerate inside the material, which is unfavorable for their uniform dispersion. As a result, the oil content of the material decreases, as seen in the sample with 3 wt% CNTs, which fails to show a better oil content. A specific analysis of the lower oil retention rate of sample CN3#-3 is as follows: The internal pore diameter of the CN3#-3 sample is relatively large, reaching 11.62 μm, much higher than the other porous samples. Since the capillary action of porous materials’ adsorption force is inversely proportional to the pore diameter, the oil retention rate of the CN3#-3 sample is lower. Overall, the oil retention rate of the porous samples prepared in this experiment is relatively high, with all samples achieving over 80%.

### 3.4. Tribological Properties of Materials

**Figure 8:**
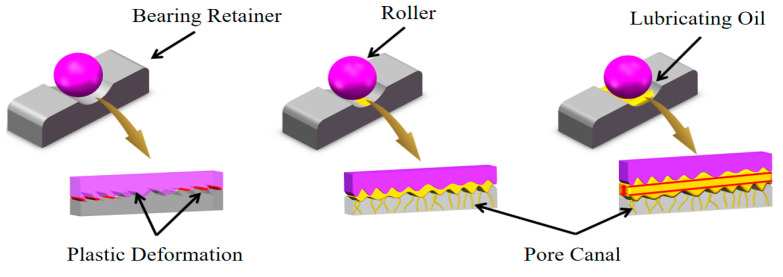
Models of Dry Friction, Boundary Lubrication, and Fluid Lubrication.

During the operation of rolling bearings, sliding contacts occur at two primary interfaces: between the cage and the rolling elements, and between the inner and outer rings. Based on the lubrication condition of the interacting surfaces, these frictional regimes are commonly categorized into dry friction, boundary friction, and fluid friction, as shown in Figure 8.

**Figure 9:**
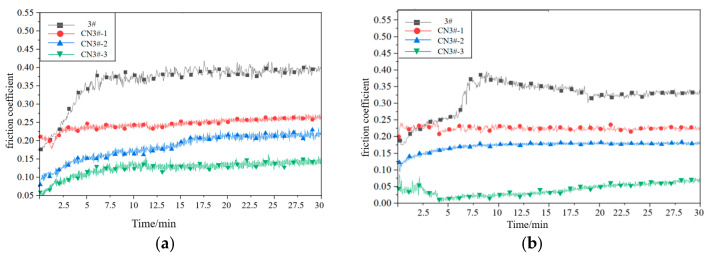
Friction Coefficient Curves of Various Samples under Dry Friction Conditions at Room Temperature (**a**) Before Heat Treatment; (**b**) After Heat Treatment.

In fluid friction, a continuous oil film separates the friction surfaces, preventing direct contact and providing optimal lubrication. However, under extreme conditions—such as in aerospace bearings subjected to ultra-high vacuum, severe temperature variations, and frequent start-stop cycles—lubricant consumption increases significantly, potentially leading to dry friction. Therefore, this study focuses on the tribological performance of porous materials under such demanding. The friction coefficient curves corresponding to the experimental samples under dry friction conditions at room temperature are shown in Figure 9 (The curve shown represents the results of experiments).

**Figure 10:**
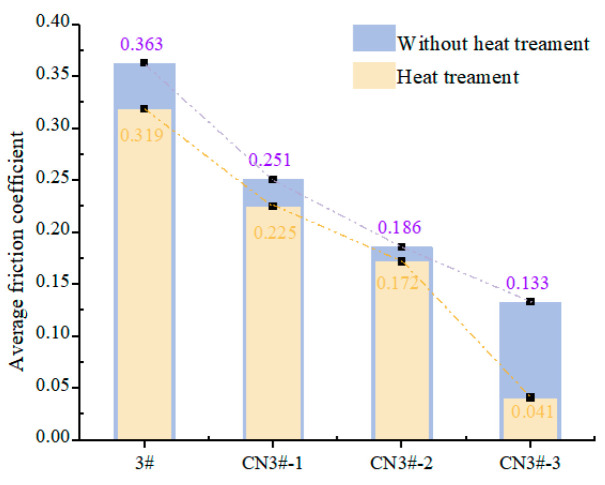
Mean friction coefficient under dry sliding.

As can be seen from Figure 9 and Figure 10, under dry friction conditions at room temperature, the CNTs-reinforced PEEK porous material exhibits a significantly lower friction coefficient compared to PEEK samples without CNTs reinforcement. Relative to pure PEEK (sample 3#), the CNTs-reinforced samples demonstrate a friction coefficient reduction of 26.4–63.4%, with the coefficient showing a negative correlation with CNTs content. Moreover, the time-dependent wear behavior of CNTs-containing porous materials is markedly more stable than that of unreinforced PEEK samples. These results indicate that incorporating CNTs effectively enhances material properties, endowing the composite with superior dry friction performance.

The friction coefficients of CNTs-reinforced PEEK porous composites exhibit a declining trend. Notably, sample CN3#-3 shows a reduction from 0.133 to 0.041 (a 69.2% decrease), while CN3#-2 displays the smallest improvement (7.5%) yet still demonstrates enhanced performance. Overall, the friction coefficient curves remain stable, suggesting that the heat treatment process contributes to improved dry friction characteristics of PEEK-based porous materials.

The formation and accumulation of fine wear debris are significant factors contributing to the instability of the wear process, particularly for porous materials. The accumulation of debris causes the pore structure to close, restricting the release and recovery of lubricating oil. The application of the heat treatment process not only effectively reduces the likelihood of wear debris formation but also ensures the self-lubricating performance of porous materials under poor oil lubrication conditions. This is the key factor that leads to the further reduction in the friction coefficient and the smoother wear process of CNTs-containing samples after heat treatment.

The friction and wear performance observed at room temperature may not accurately represent the material’s high-temperature tribological behavior. Therefore, results obtained under ambient conditions cannot reliably predict the actual service performance of the CNTs-reinforced PEEK porous composites. The dry friction process at high temperatures is more complex and places higher demands on the material’s service performance. Therefore, studying the frictional properties of CNTs-reinforced PEEK-based porous composites under high-temperature conditions aims to reveal the performance differences in PEEK-based porous materials at different temperatures.

**Figure 11:**
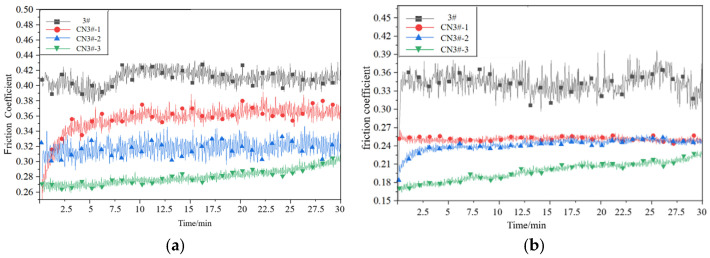
Friction coefficient curves of each sample under high-temperature dry friction conditions (**a**) Before Heat Treatment; (**b**) After Heat Treatment.

**Figure 12:**
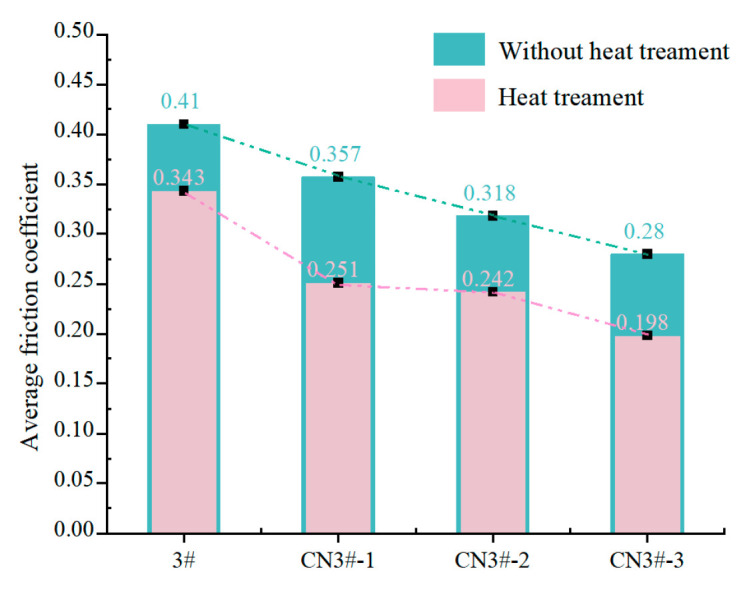
Average friction coefficient of each sample under high-temperature dry friction conditions.

It can be seen from Figure 11 and Figure 12, compared with room-temperature dry friction tests, the high-temperature dry friction tests generally exhibit higher friction coefficients. This is primarily because intense friction causes continuous temperature to rise in local areas, leading to frequent adhesive phenomena during the friction process, which consequently increases local friction coefficients.

After adding CNTs under high-temperature dry friction, the friction coefficient of the PEEK-based porous composites still shows a decreasing trend, confirming that the inherent lubricity of CNTs plays a crucial role in dry friction. The friction coefficient curve for the CNTs-containing heat-treated samples is much smoother, indicating that the material’s wear process is more stable. This suggests that heat treatment stabilizes the wear process, reducing the probability of fine debris formation under high temperatures, which in turn stabilizes the friction coefficient below 0.30. The reason for this is that after heat treatment, the crystallinity of the material’s surface layer increases, significantly improving the wear resistance of the porous PEEK material. This confirms that heat treatment optimizes the surface characteristics or lubrication mechanism under normal conditions, thereby reducing friction losses. The heat-treated samples exhibit a smaller increase in friction coefficient under high-temperature dry friction, indicating that heat treatment enhances the sample’s high-temperature stability.

**Figure 13:**
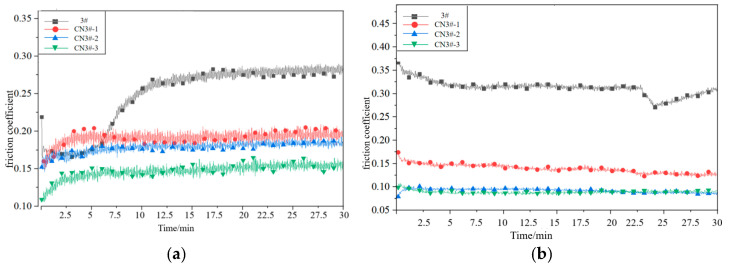
Friction coefficient curves of each sample under high-temperature, Boundary lubrication conditions (**a**) Before Heat Treatment; (**b**) After Heat Treatment.

**Figure 14:**
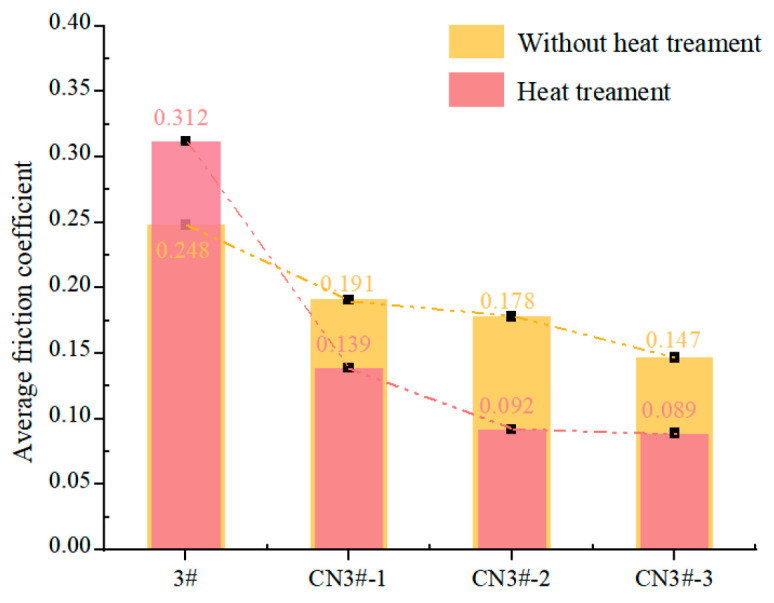
Average friction coefficient of each sample under high-temperature, Boundary lubrication conditions.

To better simulate the “insufficient lubrication” scenario that may occur in practice, high-temperature, oil-poor friction experiments were performed on both heat-treated and non-heat-treated CNTs-reinforced PEEK porous materials (Figure 13 and Figure 14).

**Figure 15:**
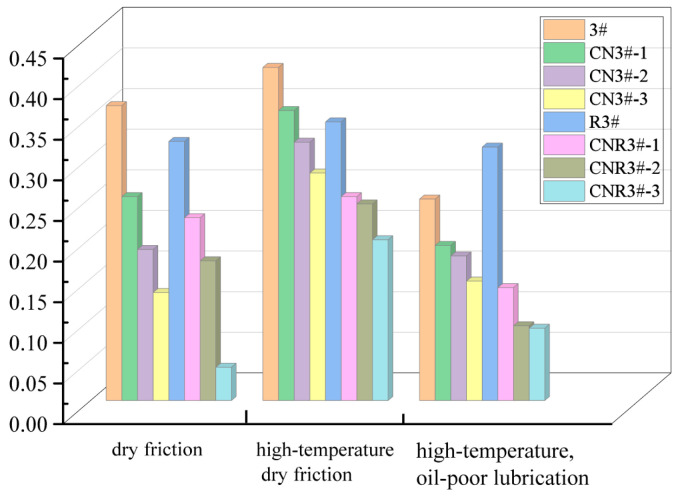
Overall average friction coefficient of the samples.

From the friction coefficient curves in Figure 13 and Figure 14, it is evident that under high-temperature, oil-poor lubrication conditions, the friction coefficient of the CNTs-enhanced PEEK-based porous composite samples is much lower than that of the PEEK samples without the CNTs reinforcement. Moreover, as the CNTs content increases, the friction coefficient decreases. This indicates that the oil-poor condition significantly improved the friction performance of the materials. All samples showed a significantly lower friction coefficient under high-temperature, oil-poor conditions compared to high-temperature dry friction. The friction coefficient of PEEK sample (3#) decreased by 25.8%, while the CN3#-2 sample showed a decrease of 48.3%. This result can be attributed to two main factors: On one hand, the trace amounts of lubricating oil at the friction interface form a boundary lubrication film, reducing direct contact. On the other hand, the cross-linking effect of CNTs within the PEEK matrix enhances the connectivity of the porous structure, facilitating the migration of lubricating oil. This confirms that CNTs-reinforced porous samples still possess a stable release and recovery function for lubricating media, even under high-temperature conditions (Figure 15).

## 4. Conclusions

This study systematically investigated the effects of carbon nanotube (CNT) content and post-processing heat treatment on the microstructure and tribological properties of PEEK-based porous composites. The main findings are summarized as follows:

(1) The incorporation of CNTs effectively modulated the pore architecture of PEEK, significantly increasing both average pore size and overall porosity. This structural optimization remarkably enhanced the oil absorption and retention capabilities of the materials, with the oil retention rate consistently exceeding 80%.
(2) CNT-reinforced PEEK porous composites demonstrated superior tribological performance across multiple testing environments. The most significant improvement was observed under room-temperature dry sliding conditions, where the friction coefficient was reduced by 26.4% to 63.4% compared to unreinforced porous PEEK. The composites also exhibited excellent wear resistance and operational stability.
(3) Heat treatment further enhanced the performance of CNT-reinforced composites, substantially improving their friction behavior and thermal stability. Under high-temperature dry friction conditions, the friction coefficient remained stable below 0.30, indicating the effective slow-release and recovery functionality of lubricating media even in elevated-temperature environments.

Study Limitations and Future Perspectives:

While this study demonstrates the potential of CNT-reinforced porous PEEK composites, several limitations should be acknowledged. The current work focused primarily on feasibility verification, thus comprehensive parameter optimization and in-depth mechanistic studies were not fully explored. Specifically, the tribological tests were conducted under limited conditions, and detailed analysis of wear mechanisms was not included.

Future research should focus on the following directions:

1. Systematic investigation of tribological behavior under varying operational parameters (load, velocity, temperature). 2. Quantitative analysis of the oil release kinetics and lubrication mechanisms during dynamic operation. 3. Detailed characterization of wear mechanisms through advanced microscopy and spectroscopy techniques. 4. Optimization of the heat treatment process to achieve better balance between crystallinity control and CNT stability.

## Figures and Tables

**Table 1:** Main Parameters of CNTs.

OD (nm)	Purity (wt%)	Length (Microns)	SSA (m^2^/g)	Ash Content (ASH) (wt%)	COOH Content (wt%)
30~50	>98%	<10	>100	<1.5	0.73

**Table 2:** Major Component Proportions and Sample Codes of Porous Materials.

Mass Ratio of PEEK to NaCl Powder	CNTs Content	Sample Identification Number	
		As-Prepared Sample	Heat-Treated Sample
1:1	0	3#	R3#
	0.5 wt%	CN3#-1	CNR3#-1
	1 wt%	CN3#-2	CNR3#-2
	3 wt%	CN3#-3	CNR3#-3

**Table 3:** Temperature Settings for Each Heating Zone of the Twin-Screw Extruder.

Heating Zone	Zone 1	Zone 2	Zone 3	Zone 4	Zone 5	Zone 6	Zone 7
PEEK/CNTs/NaCl	331	334	336	337	343	341	334

**Table 4:** Characterization test equipment.

Equipment Name	Type-Specification	Manufacturer	Main Technical
FESEM	JSM-IT800	Japanese electronics company	Resolution: 0.7 nm
			Acceleration voltage: 0.01~30 kV
			Stroke: X direction 70 mm, Y direction 50 mm, Z direction 1.5~41 mm
Mercury Porosimeter	Autopore IV 9500	Micromeritics (USA)	Accuracy: 50~1 × 10^6^ A
			High-Pressure Accuracy: 1 Psi
			Pore Size Measurement Range: 5 × 10^−4^~1 × 10^3^ μm
LSCM	LSM800	Carl Zeiss (Germany)	Accuracy: 0.2 μm
			Repeatability: <1 μm
			Absolute Accuracy: ±5 μm
			Image Resolution: 4 × 1 to 6144 × 6144 pixels
			Optical Resolution: X/Y: 120 nm; Z: 1 nm

## Data Availability

Data are contained within the article.
